# Reanalysis of updated mortality among vinyl and polyvinyl chloride workers: Confirmation of historical evidence and new findings

**DOI:** 10.1186/1471-2458-8-21

**Published:** 2008-01-22

**Authors:** Valerio Gennaro, Marcello Ceppi, Paolo Crosignani, Fabio Montanaro

**Affiliations:** 1 Epidemiology and Prevention Dept., National Cancer Research Institute (IST), Genova, Italy; 2 Cancer Registry and Environmental Epidemiology, National Cancer Institute (INT), Milano, Italy

## Abstract

**Background:**

The production of vinyl chloride (VC) and polyvinyl chloride (PVC) involves the use of various chemicals, some known to be toxic and potentially or definitely carcinogenic. The related potential risk often has not been properly investigated. Updated cancer mortality among different subgroups of workers employed in a VC-PVC production plant located in Porto Marghera (Italy) was re-analyzed using an internal reference group of workers with low (or null) exposure to VC.

**Methods:**

Mortality of 1658 male workers was analyzed by Poisson regression. Relative risks (RRs) and 95% confidence intervals (CIs) for blue collar workers and their specific subgroups of PVC baggers, PVC compound, autoclave and other blue collar workers were calculated using technicians and clerks as an internal reference group. The follow-up covered the period 1972–1999.

**Results:**

Significantly increased mortality rates were observed for all causes of death among the whole blue collar workforce (RR = 1.55; 95% CI = 1.03–2.35; 229 deaths), PVC baggers (1.72; 95% CI = 1.04–2.83; 49 deaths) and PVC compound workers (1.71; 95% CI = 1.09–2.67; 72 deaths). Liver cancer, including angiosarcoma, was increased among autoclave workers (9.57; 95% CI = 3.71–24.68; 7 deaths) and cardiovascular diseases among PVC baggers (2.25; 95% CI = 1.08–4.70; 12 deaths). Hemolymphopoietic system tumors, leukemias and lymphomas prevalently, were found only among exposed workers, with 4, 4 and 6 deaths observed among PVC baggers, PVC compound and other blue collar workers, respectively. An excess of lung cancer was found among PVC baggers.

**Conclusion:**

This cohort analysis, based on internal comparison, confirmed previously reported specific risk excesses for liver tumors and liver cirrhosis among autoclave workers and for lung cancer among PVC baggers, and revealed PVC compound workers as a possible new at risk group for all causes, all tumors and for liver and lung tumors. In conclusion, RRs for all causes of death and all tumors were increased among all blue collar workers.

## Background

The production of vinyl chloride (VC) and polyvinyl chloride (PVC) entails the use of various chemicals (Table 1)[1], some of which are known to be toxic and potentially or definitely carcinogenic [2]. Indeed, a number of these compounds have been reported to induce cancer in the liver (prevalently hepatic angiosarcoma) [3-5], the hemolymphopoietic system (leukemias and lymphomas), lung and brain [3,4].

**Table 1:** Provisional list of chemicals [CAS number] used in the production of VC and PVC and related products

Acetates	Alcohol	Sodium hydroxide [1310-73-2]	Phosgene [75-44-5]
Acetylene [74-86-2]	Chlorofluoromethane [593-70-4]	Sulphates [EDF-078]	Plasticizers
Acetone cyanhydrin [75-86-5]	Ammonia [7664-41-7]	Ethylene [74-85-1]	Stabilizers
Acids:	Asbestos [1332-21-4]	Hexane [110-54-3]	Tedimon 31 [9016-87-9]
acetic [64-19-7]	Caprolactam [105-60-2]	Dichloroethane [1300-21-6]	Terephthalates
hydrocyanic [74-90-8]	Chlorine [7782-50-5]	Diphenylmethane diisocyanate	Vinyl chloride [75-01-4]
hydrochloric [7647-01-0]	Dioxide	[101-68-8]	Polyvinyl chloride [9002-86-2]:
fluorhydric [7664-39-3]	Fluorides [16984-48-8]	Tetrachloroethane [79-34-5]	Granules, compound, suspension, copolymers, emulsion.
nitric [7697-37-2]	Hydramine	Tetrachloroethylene [127-18-4]	
sulphuric [7664-93-9]	Hydrogen [1333-74-0]	Trichloroethylene [79-01-6]	
hypochlorous [7790-92-3]	Sodium cyanide [143-33-9]	Toluene diisocyanate [26471-62-5]	
Acrylonitrile [107-13-1]	Potassium cyanide [151-50-8]	Petrol	

Numerous investigators have analyzed a cohort of Italian VC-PVC workers, who had been the subject of a large class-action lawsuit [1]. One study on this cohort, updated in 1995, found an elevated death risk for all causes among the whole group of exposed (i.e., blue collar) workers, and specific death risks among specific subgroups, such as liver cancer and liver cirrhosis among autoclave workers and lung cancer among PVC baggers[6]. On the contrary, other analyses on the same workers revealed a statistically significant reduced mortality from all deaths combined and a not statistically significant reduction of mortality from all tumors among the whole cohort [1,7,8]. Since these latter studies investigated the entire cohort – including both exposed and non-exposed workers – and used the general population of the Region where the plant is located (Veneto) as a reference, related analyses may well have hidden some health risk excesses. For this reason, the updated mortality of the same cohort was reanalyzed using an internal comparison group of non-exposed workers in order to describe the pattern of diseases specific for each subgroup of exposed workers.

## Methods

### Population under study

The present update of the follow-up included 1658 employees (42253 person-years) who had worked in a large petrochemical plant located in Porto Marghera (Venezia, Italy) for any period of time between July 1950 and July 1985. The follow-up period extended from January 1972 to December 1999; 248 deaths were registered. The classification and coding of previous studies were maintained, both for job category (28 plant sectors, used as surrogates of homogenous exposures) [1,7] and for cause of death (ICD-9th revision [9]).

Workers were classified according to their job categories in four large, but reasonably homogeneous, groups: *autoclave workers *(n = 210, 5446 person-years), *PVC baggers *(n = 198, 4978 person-years), *PVC compound workers *(i.e., workers employed in the production of PVC granules from resins, n = 404, 10310 person-years) and *technicians and clerks *(n = 202, 5303 person-years). The above workers comprised 61% of the total workforce. An additional, more heterogeneous group, categorized as *other blue collar workers *(n = 644; 16216 person-years), included the plant employees in the remaining 24 sectors.

As *technicians and clerks*, thanks to environmental workplace sampling, were found in a previous study to be a group with low (or null) exposure to toxic and carcinogenic agents, they were used as an internal reference in the present analysis [10].

This reanalysis considered all causes of death previously reported as being associated to an increased risk [3,4].

An analysis of changes in job category revealed that only 15 workers transferred from an exposed to an unexposed category, and that only nine moved from an unexposed to an exposed category. Overall, 81% of workers never changed their initial occupation. For this reason, the job category at hiring was used to split the workforce into different categories.

### Statistical Analysis

Considering overall mortality among all exposed subjects (i.e., the total blue collar workforce), the study had an estimated power of 0.80 to detect a univariate relative risk (RR) of 1.50 as significantly increased at a 0.05 level (two-sided).

An internal comparison, using the above mentioned reference group, was carried out through a Poisson regression model for main causes of death.

Potential confounding factors considered in the analysis were age (five-year classes, 25–75+), calendar period of follow-up (1972–1974, 1975–1999 by five-year periods), age at hiring (2 levels: before age 25, 25+), year of hiring (before 1973, 1973 or later), duration of exposure (i.e., duration of employment; 3 levels: 0–4; 5–9; 10+), and latency (i.e., time since first hiring; 2 levels: 0–9; 10+). Data were grouped into cells according to the level of the explanatory variables described above.

The model used was:

*ln(Ok) = ln(Ek) + α + β i****X****i *+ *ε*_*k*_

where *O*_*k *_are the observed deaths for a specific cause in the *k*^*th *^cell, and *E*_*k *_are the expected deaths in the *k*^*th *^cell according to age, calendar year and cause specific mortality rates of the Italian male population (*ln() *are their natural logarithms); *exp(α) *is the Standardized Mortality Ratio (SMR) compared to the Italian population for persons falling in the baseline category (i.e., with all the variables set at the reference level); ***X***_*i *_is a matrix of indicator variables that specify the levels of the explanatory variables defining each cell; *β*_*i *_is a vector of unknown parameters to be estimated from the data, such that *exp(β*_*i*_*) *represents the Relative Risk (RR) related to each explanatory variable with respect to its own reference level; *ε*_*k *_is the random error. Each job category RR estimated by this model represents the ratio between the SMR of the exposed group and the SMR of the unexposed one for each specific cause of death. As suggested by Breslow and Day, the linkage of the method of indirect standardization to the multivariate regression analysis improves the efficiency of the statistical modeling [11].

The impact of the inclusion of each possible confounder in the regression models was tested through the likelihood-ratio test and by evaluating the change in the estimated RRs and their standard errors.

In order to avoid biased estimation of the standard errors of the parameters and to improve the goodness-of-fit of the selected model, when the ratio between the residual deviance (DV) and its degrees of freedom (DF) was far from 1 (i.e., in presence of over- or underdispersion), we refitted the model by introducing the following scale parameter:

*DV*_*MAX*_*/DF*_*MAX*_

where *DV*_*MAX *_and *DF*_*MAX *_were obtained by fitting a maximal model, i.e., a model with a high level of interactions, as suggested by Aitkin et al. [12].

Due to the low number of cases of liver and brain tumors and for liver cirrhosis, Bayesian estimates of the mean of the posterior distribution of RR (RR_pm_) for these diseases were obtained by means of the Monte Carlo – Markov Chain method [13].

Confidence intervals at the arbitrary levels of both 95% (95% CI) and 95% Bayesian credibility interval (95% BCI) between the 2.5^th ^and 97.5^th ^percentile of the posterior distribution of RR, were estimated as appropriate. To assess the goodness-of-fit of the statistical models, standard diagnostic procedures were performed. Poisson models were fitted to the data using STATA statistical software [14].

The attributable number of deaths (AD) among exposed workers was estimated according to the following equation:

$$AD=d\cdot \left(\frac{RR_{e}-1}{RR_{e}}\right)$$

where *RR*_*e *_is the RR of the exposed (sub)group and *d *is the number of observed deaths.

## Results

The numbers of workers, mean age at hiring and person-years by age for each subgroup of workers (*autoclave workers*, *PVC baggers*, *PVC compound workers*, *other blue collar workers*, and *technicians and clerks*) are reported in table 2.

**Table 2:** Number of workers, mean age at hiring and standard deviation (SD), person-years (PY) by age class for each group of workers

	**Technicians and clerks^1^**	**Autoclave workers**	**PVC Baggers**	**PVC Compound workers**	**Other blue collar workers**	**Total blue collar workforce^2^**	**Total**
**Number of workers**	202	210	198	404	644	1456	1658

**Mean age at hire (SD)**	27.4 (7.1)	26.1 (5.2)	31.7 (7.5)	29.5 (7.3)	29.5 (8.2)	29.3 (7.6)	29.1 (7.6)

**PY age <30**	266	325	79	333	897	1634	1900
**PY age 30–44**	2025	2298	1237	3388	5938	12861	14886
**PY age 45–59**	2283	2312	2483	4879	6966	16640	18923
**PY age >59**	729	511	1179	1710	2415	5815	6544

**Total PY**	5303	5446	4978	10310	16216	36950	42253

^1 ^Reference group

^2^Total blue collar workforce = Autoclave workers + PVC Compound workers + PVC Baggers + Other blue collar workers.

Numbers of observed deaths (Obs), RRs and 95% CIs (or RR_pm_s and 95% BCIs, as appropriate [see *Methods*]) by job category using *technicians and clerks *as a reference are shown in table 3.

**Table 3:** Number of observed deaths (Obs), relative risk (RR)^1 ^and 95% confidence intervals (95% CI)^2 ^obtained by Poisson regression^3^ for main causes and workers' subgroups

**Causes of death (ICD IX)**		***Technicians & Clerks (Reference)***	**Autoclave workers**	**PVC Baggers**	**PVC Compound workers **	**Other blue collar workers**	**Total blue collar workforce**^4^
**All causes (000–999)**	***Obs***	***19***	25	49	72	83	229
	***RR***		1.58*	**1.72**	**1.71**	1.41	**1.55**
	***(95% CI)***		(0.92–2.71)	**(1.04–2.83)**	**(1.09–2.67)**	(0.91–2.19)	**(1.03–2.35)**

**All tumors (140–208)**	***Obs***	***11***	14	23	38	43	118
	***RR***		1.60	1.53	1.59	1.26	1.42
	***(95% CI)***		(0.74–3.45)	(0.73–3.18)	(0.83–3.04)	(0.67–2.39)	(0.79–2.57)

**Lung tumor (162)**	***Obs***	***3***	2	11	12	12	37
	***RR***		0.94	3.13	1.90	1.23	1.60
	***(95% CI)***		(0.19–4.61)	(0.96–10.28)	(0.62–5.80)	(0.40–3.75)	(0.54–4.75)

**Lymphoid and hematopoietic tumor (200–208)**	***Obs***	***0***	-	4	4	6	14
	***RR***						
	***(95% CI)***						

**Brain tumor (191)**	***Obs***	***1***	-	-	2	-	2
	***RR***_pm_				1.11		0.36
	***(95% BCI)***				(0.10–13.03)		(0.03–4.30)

**Liver tumor(155)**	***Obs***	***1***	7	1	5	3	16
	***RR***_pm_		**9.57**	0.82	2.46	1.17	2.39
	***(95% BCI)***		**(3.71–24.68)**	(0.23–2.93)	(0.94–6.42)	(0.47–2.90)	(0.63–9.11)

**Liver cirrhosis (571)**	***Obs***	***2***	5	3	5	5	18
	***RR***_pm_		2.80	1.03	1.10	0.88	1.17
	***(95% BCI)***		(0.90–8.76)	(0.28–3.78)	(0.36–3.39)	(0.29–2.67)	(0.41–3.38)

**Cardiovascular diseases (390–452)**	***Obs***	***3***	3	12	9	18	42
	***RR***		1.19	**2.25**	1.29	1.89	1.68
	***(95% CI)***		(0.49–2.86)	**(1.08–4.70)**	(0.63–2.64)	(0.97–3.67)	(0.86–3.30)

^1 ^RRpm: mean of the posterior distribution of RR

^2^95% BCI: 95% Bayesian credibility interval between the 2.5^th ^and 97.5^th ^centiles of the posterior distribution of RR.

^3^RRs and RR_pm_s are adjusted by age, calendar period, employment duration and latency.

^4^Total blue collar workforce = *Autoclave workers *+ *PVC Compound workers *+ *PVC Baggers *+ *Other blue collar workers*.

The total blue collar workforce (last column of tab. 3) experienced significantly increased mortality from all causes (RR = 1.55, 95% CI 1.03–2.35; 229 deaths); RRs for all tumors and for each specific cause of death considered were increased, but not significantly.

The comparison of each subgroup of blue collar workers with the internal reference revealed different risk patterns. In fact, RR estimates were significantly increased for liver cancer (RR_pm _= 9.57, 95% CI: 3.71–24.68) among *autoclave workers*, for all causes (RR = 1.72, 95% CI: 1.04–2.83) and cardiovascular diseases (RR = 2.25, 95% CI: 1.08–4.70) among *PVC baggers*, and for all causes (RR = 1.71, 95%CI: 1.09–2.67) among *PVC compound workers*.

Even among *other blue collar workers *excesses (not statistically significant) were observed for each considered cause of death, except for liver cirrhosis.

Fourteen deaths from hemolymphopoietic system tumors were detected among blue collar workers, but none were seen in the referent group (or among *autoclave workers*), thereby preventing us from estimating related RRs. As an indicative risk for these tumors, univariate SMRs compared to the Italian population were estimated. A significantly increased SMR was observed among *PVC baggers *(SMR = 3.77; 95% CI: 1.03–9.66) and among the total blue collar workforce (SMR = 2.27; 95% CI: 1.24–2.81), while *PVC compound workers *and *other blue collar workers *showed SMRs equal to 2.26 and 2.29, respectively (not statistically significant).

Some of the analyzed causes of death showed well increased RR estimates, at a borderline significance at two-tailed 95% significance level. In particular, most worthy of attention were the higher than two-fold increases found for liver cirrhosis (RR_pm _= 2.8) among *autoclave workers*, for lung cancer (RR = 3.13) among *PVC baggers *and for liver cancer (RR_pm _= 2.46) among *PVC compound workers*. These increases would have been statistically significant at an one-tailed 95% significance level.

As a final point, 81.3 deaths (out of 229 observed) from all causes and 34.9 deaths (out of 118) from all tumors were estimated to be attributable to blue collar workers' activity in this plant.

## Discussion

Following an extensive review and evaluation of previous studies, the IARC, first in 1979 and then in 1987 [3,4], provided sound evidence for a causal association between VC-PVC exposure and liver cancer, as well as tumors of the brain, lung and hemolymphopoietic system. Despite such an authoritative stance on the issue, a good share of epidemiological literature continues to consider liver angiosarcoma as the only proven tumor caused by working in this petrochemical sector, mainly because RRs for this rare tumor have been found to be statistically elevated in almost all published studies [5,8,15-18]. Excesses for hepatic tumors other than liver angiosarcoma, when detected, have been considered as misclassifications of angiosarcoma [15,16]. Risks for other neoplastic and non-neoplastic diseases have been often ignored, with the exception of some findings for brain [17] and hemolymphopoietic system cancer [18]. Nevertheless, excesses for lung cancer have recently been re-detected in specific subgroups of workers exposed to PVC dust employed in the same Italian plant [8,19], and our previous studies [6] found excesses of liver tumors and liver cirrhosis among *autoclave workers *and of lung cancer among *PVC baggers*.

In an attempt to clarify these contradictory results, in which the healthy worker effect (HWE) likely played an important role, the same cohort of VC-PVC workers was reanalyzed using an internal comparison group constituted by unexposed (or less exposed) workers, instead of an external population based on general regional or national figures. External comparison is probably one of the major causes of biased results, mainly due to the evaluation of very different populations (likely very healthy workers vs. the mix of healthy and unhealthy people in the general population). Consequently, the HWE may lead to a relative underestimation of the true mortality risks among exposed workers.

Our reanalysis has revealed a significantly elevated risk for all causes of death among exposed blue collar workers compared to unexposed *technicians and clerks*. Specifically, increased mortality from both liver tumors (RR_pm _= 9.57; *p *< 0.05) and liver cirrhosis (RR_pm _= 2.80; *p *< 0.10) was observed among *autoclave workers*, where both an elevated risk of liver angiosarcoma [7] and the exposure to VC and PVC dusts during reactor cleaning operations have already been documented [10]. A case-control study nested in the same cohort, by holding the confounding factors constant in a logistic regression analysis, found that each extra increase of 1,000 ppm × years of VC cumulative exposure increased the risk of hepatocellular carcinoma by 71% (OR = 1.71; 95% CI, 1.28–2.44) and the risk of liver cirrhosis by 37% (OR = 1.37; 95% CI, 1.13–1.69) [20].

An increased mortality from all causes (RR = 1.72; *p *< 0.05) and lung cancer (RR = 3.13; *p *< 0.10) was clearly observable in *PVC baggers*. Death risk from lung cancer confirmed a recent case-control study nested in the same cohort, which found a 20% increased risk for this cause of death among *PVC baggers *for each extra year worked (OR = 1.20; 95% CI = 1.08–1.35; p = 0.0010) after adjustment for age and smoking habits [19]. In addition, previous analyses revealed a statistically significant dose-response relationship (p < 0.05) between duration of employment and lung cancer mortality, and a progressive reduction in mortality after work cessation. The risk decreased from the high value observed 5 years after work cessation (SMR = 2.4) to values lower than unity after 15–20 years [6]. This trend reflected what was observed by Doll and Peto among former smokers after smoking cessation [21], and also by Mastrangelo et al., who reported that "*In our [PVC] baggers, recent rather than distant exposure to PVC dust had the most profound effects on lung cancer risk*" [19].

The main task of *PVC compound workers *was to mix granular PVC with many other likely toxic and carcinogenic chemical agents (Tab. 1), whose characteristics differed according to the final PVC-based product [22]. Workers performing this task should be considered as a new at risk group, since they showed an increased risk of death from all causes (RR = 1.71; *p *< 0.05) and from liver cancer (RR_pm _= 2.46; *p *< 0.10), and RRs for all tumors combined and lung cancer were increased too, even though increase was not statistically significant.

*Other blue collar workers *showed excess (not statistically significant) risks of death for nearly all causes considered. This result suggests that high health risks occurred among all workers in this category, in addition to those usually considered at risk (i.e., *autoclave workers *and *PVC baggers*).

Deaths from hemolymphopoietic system tumors (14 cases) occurred only among exposed workers (*PVC baggers*, *PVC compound workers*, *other blue collar workers*), preventing any estimate of RR. For this reason, SMRs were calculated and revealed significantly increased values among the total blue collar workforce and specifically among *PVC baggers*, while *PVC compound workers *and *other blue collar workers *showed more than two-fold increased SMRs (not statistically significant). These findings support the causal association already reported in the scientific literature between occupational exposure and hemolymphopoietic system tumors [3,4].

### Attributable number of deaths

The total number of deaths attributable to employment as a blue collar worker in a VC-PVC production plant was estimated to be 83.2 (out of 160) and 81.3 (out of 229) when the follow-up was updated to 1995 and 1999, respectively [23]. The consistency of these values is evidence of the reliability of results.

The longer follow-up did not lead to an increase in the total number of attributable deaths. Actually, while the RR was estimated to be 2.09 after the previous update (1972–1995), it was estimated to be 0.97 for the most recent 5-year period (1995–1999), suggesting that the effect of the exposure to noxious substances – or exposure itself – may have been greater in the past and lower in more recent years. On the other hand, as expected for diseases with a long latency (i.e., tumors), attributable deaths from all tumors increased from 28.1 to 34.9 when the duration of follow-up was increased.

### Limitations, strengths and remarkable results

The present epidemiological study, like many other studies on occupational cohorts, lacked information about measures of individual occupational exposures over time during the employment period. Job category and length of employment were used as surrogate indicators of exposure type and cumulative exposure, respectively. Two studies on the same cohort already cited [19,20] revealed a drawback in the cohort database stemming from a misclassification of both exposure and disease (information bias). In any case, such a misclassification could have led to an underestimation of the risk among exposed workers. A lack of fund prevented a more recent follow-up update, nevertheless the study had a power large enough to detect significant risks. Finally, it could be prudent to bear in mind that the study suffered from some limitation concerning the control of possible confounders, even if it unlikely could have lead to a significant modification of the results, as it will be made clearer afterward.

Despite these limitations, this analysis was, in our opinion, grounded on a reasonable approach and achieved some noteworthy results. First of all, the present reanalysis sought to reduce the comparison bias by using as a reference group – instead of the general population – a working population with limited or null exposure to occupational carcinogenic agents and with hiring criteria and duration of employment similar to those of the exposed group(s) under study. In addition, we tried to avoid another possible source of underestimation of the true risk, i.e. the inclusion of unexposed (or less exposed) workers in the exposed group (misclassification of exposure). In this way, the HWE and the related underestimation of the health risks should have been eliminated or at least controlled. Even if it was not possible to assess whether and how *technicians and clerks *might differ from the exposed workers other then the exposures of interest, the comparison seemed appropriate, and the socio-economic status of the exposed and unexposed workers should be fairly comparable. For this reason and as it was found to be at low (or null) exposure to toxic and carcinogenic agents [10], this group of workers (i.e., technicians and clerks) was the best available comparison group, while *other blue collar workers *included workers employed in different tasks not specialized and/or not confined in a specific workplace, such maintenance staff, cleaning operators, and other workers that handled, or were exposed to, most of the toxic substances listed in table 1. A recent study tried to quantify the possible amount of the bias due to the joint effect of the dilution effect (DE) and the comparison bias (CB) in occupational cohort studies. DE and CB respectively occur when exposed and unexposed workers in a cohort are pooled together and an internal referent group is not used. The study confirmed that *"results from occupational cohort studies without internal referent groups may be unreliable" *[24]. Then, internal comparison and separating blue collar workers into subgroups with homogenous exposure reduced the possible DE and resulting estimates were likely not biased by confounding due to smoking or to other factors. As suggested by "good epidemiological practice" guidelines [25], RR estimates obtained using an internal reference are more reliable than those based upon both national and local reference, and, as it was observed, *"internal analyses of "dose-response" in cohort studies are unlikely to be seriously confounded by smoking habits" *[26]. Within the same cohort analyzed in the present study, Mastrangelo et al. [19] found a lung cancer risk among baggers comparable to our estimates, but using a case-control approach, that by definition takes smoking (as other confounders) into account. Moreover, a clear dose-response relationship between length of exposure and lung cancer was detected among PVC bagger workers. In fact, not exposed workers and PVC bagger workers with a length of employment – used as a proxy of length of exposure – of 0–5, 6–10 and 10+ years, showed the following SMRs: 64.79, 108.35, 216.45 and 355.86, respectively. As this trend (*p *= 0.054) was observed among workers belonging to the same job category, it was likely unaffected by confounding effects, including those generated by smoking status. Finally, applying the Axelson approach to indirect adjustment for a confounder, it was clear that smoking unlikely played a major role when lung cancer RR was greater than 1.66 [27,28].

The two procedures used in this study (i.e. internal comparison and separating blue collar workers into subgroups with homogenous exposure) revealed the pattern of diseases, likely due to exposures to specific occupational risk factors, typical of each subgroup of workers.

## Conclusion

The present study confirmed the elevated risks for specific tumors, namely liver, hemolymphopoietic system and lung cancers, previously observed by the scientific community (above all, the IARC [2-4]). In particular, two job categories, *autoclave workers *and *PVC baggers*, were confirmed to be at risk for liver diseases and lung cancer, respectively, and another category, *PVC compound workers*, was found to be likely at risk for liver and lung tumors. Moreover, mortality risk from neoplastic and non-neoplastic diseases was shown to be high among all blue collar workers. These findings confirm that differently exposed workers could become ill from different diseases.

The simple and rigorous approach applied in this study aimed to offset main biases and distortions that usually affect occupational cohort studies, and the new evidence emerging from this reanalysis confirms the already documented health risks. We believe that this methodology could be used for the analysis of several other cohort studies.

## Competing interests

During the class action lawsuit against the Marghera petrochemical plant, VG and PC were appointed as technical consultants by the plaintiffs Greenpeace and Medicina Democratica, respectively. MC and FM have no competing interests to declare. The study was not financially funded.

## Authors' contributions

VG conceived the study and supervised all aspects of its implementation. MC performed the statistical analyses and wrote related sections. FM participated in the design of the study, assisted with the analyses and led the writing. PC revised all aspects of the study and helped to draft the manuscript. All authors helped to conceptualize ideas and interpret findings. All authors read and approved the final manuscript.

## Pre-publication history

The pre-publication history for this paper can be accessed here:


